# Comparison of Automated Point-of-Care Gram Stainer (PoCGS^®^) and Manual Staining

**DOI:** 10.3390/diagnostics15091137

**Published:** 2025-04-29

**Authors:** Goh Ohji, Kenichiro Ohnuma, Kei Furui Ebisawa, Mari Kusuki, Shunkichi Ikegaki, Hiroaki Ozaki, Reiichi Ariizumi, Masakazu Nakajima, Makoto Taketani

**Affiliations:** 1 Division of Infectious Disease Therapeutics, Department of Infectious Disease, Kobe University Graduate School of Medicine, 7-5-2, Kusunokicho, Chuoku, Kobe 6500017, Japan; k.f.ebisawa@gmail.com (K.F.E.); ikegaki@port.kobe-u.ac.jp (S.I.); 2 Department of Clinical Laboratory, Kobe University Hospital, 7-5-2, Kusunokicho, Chuoku, Kobe 6500017, Japan; onumak@med.kobe-u.ac.jp (K.O.); hyakuta@med.kobe-u.ac.jp (M.K.); 3 CarbGeM Inc., 5-13, 1chome, Jinnan, Shibuyaku 1500041, Japan; hiroaki.ozaki@carbgem.com (H.O.); masakazu.nakajima@carbgem.com (M.N.); makoto.taketani@carbgem.com (M.T.)

**Keywords:** Gram stain, point of care, emergency department, intensive care unit, automated Gram stainer

## Abstract

**Background/Objectives:** Gram staining is an essential diagnostic technique used for the rapid identification of bacterial and fungal infections, playing a pivotal role in clinical decision-making, especially in point-of-care (POC) settings. Manual staining, while effective, is labor-intensive and prone to variability, relying heavily on the skill of laboratory personnel. Current automated Gram-staining systems are primarily designed for high-throughput laboratory environments, limiting their feasibility in decentralized healthcare settings such as emergency departments and rural clinics. This study aims to introduce and evaluate the Point-of-Care Gram Stainer (PoCGS^®^), a compact, automated device engineered for single-slide processing, addressing challenges related to portability, standardization, and efficiency in POC applications. **Methods**: The PoCGS^®^ device was developed to emulate expert manual staining techniques through features such as methanol fixation and programmable reagent application. A comparative evaluation was performed using 40 urine samples, which included both clinical and artificial specimens. These samples were processed using PoCGS^®^, manual staining by skilled experts, and manual staining by unskilled personnel. The outcomes were assessed based on microbial identification concordance, the staining uniformity, presence of artifacts, and agreement with the culture results. Statistical analyses, including agreement rates and quality scoring, were conducted to compare the performance of PoCGS^®^ against manual staining methods. **Results**: PoCGS^®^ achieved a 100% concordance rate with expert manual staining in terms of microbial identification, confirming its diagnostic accuracy. However, staining quality parameters such as the uniformity and presence of artifacts showed statistically significant differences when compared to skilled and unskilled personnel. Despite these limitations, PoCGS^®^ demonstrated a comparable performance regarding artifact reduction and agreement with the culture results, indicating its potential utility in POC environments. Challenges such as fixed processing times and limited adaptability to varying specimen characteristics were identified as areas for further improvement. **Conclusions**: The study findings suggest that PoCGS^®^ is a reliable and valuable tool for microbial identification in POC settings, with a performance comparable to skilled manual staining. Its compact design, automation, and ease of use make it particularly beneficial for resource-limited environments. Although improvements in staining uniformity and background clarity are required, PoCGS^®^ has the potential to standardize Gram staining protocols and improve diagnostic turnaround times. Future developments will focus on optimizing staining parameters and expanding its application to other clinical sample types, ensuring robustness and broader usability in diverse healthcare settings.

## 1. Introduction

Gram staining, a method devised by Hans Christian Gram in 1884, remains a cornerstone of microbiological diagnostics, facilitating the rapid classification of bacteria into Gram-positive and Gram-negative categories based on their cell wall structures, and further classifying them into bacilli and cocci based on their morphology. This technique is critical for the early identification of bacterial pathogens, guiding initial empirical therapy in clinical settings. The ability to swiftly distinguish between bacterial types enables clinicians to administer targeted antimicrobial therapy, thereby reducing morbidity and mortality associated with infectious diseases. Despite its inception over a century ago, Gram staining continues to be widely adopted due to its simplicity, cost-effectiveness, and ability to yield results within minutes. This rapid turnaround is particularly valuable in healthcare environments such as intensive care units (ICUs), emergency departments, and rural healthcare facilities with insufficient staff, where timely intervention can significantly alter patient outcomes [1,2].

The enduring relevance of Gram staining is largely attributed to its pivotal role in directing initial therapeutic decisions before more sophisticated culture-based or molecular diagnostic methods can be completed. In environments with limited resources, the technique provides an indispensable tool for guiding treatment in cases of sepsis, pneumonia, and meningitis, among other critical infections. However, the manual process of Gram staining is fraught with challenges, including a variability in results due to inconsistencies in technique and interpretation, potential contamination, and the time-intensive nature of the procedure.

Historically, manual Gram staining has been the standard approach, relying heavily on the expertise and consistency of trained laboratory personnel. This labor-intensive process involves multiple sequential steps, including fixation, staining, decolorization, and counterstaining. Each step is prone to variability depending on the technician’s skill and environmental conditions. A critical challenge associated with the manual method is the potential for human error and subjectivity in interpretation, which can lead to misdiagnosis and inappropriate treatment.

To address these limitations, Drew et al. pioneered the development of automated Gram-staining machines in the 1970s, which enhanced reproducibility and efficiency by automating the movement of slides through staining solutions [3]. These systems have been widely adopted in high-volume laboratories, where they offer advantages such as the standardization of staining protocols, a reduction in turnaround time, and the minimization of personnel workload [4]. However, their large size (533 mm W × 559 mm D × 241 mm H, as in the Previ Color Gram by bioMérieux, 376 Chemin de l‘Orme, 69280 Marcy l‘Étoile, France), high cost, and operational complexity render them unsuitable for point-of-care (POC) settings such as emergency departments, outpatient clinics, field hospitals, and resource-constrained healthcare facilities.

A significant advancement in Gram-staining methodology has been the exploration of alternative fixation techniques. Traditional heat fixation, while commonly used, is associated with several artifacts—such as ruptured red blood cells—that obscure the visualization of rare organisms. Studies by Mangels et al. and Baron et al. have highlighted the superiority of methanol fixation in preserving slide quality, maintaining cellular morphology, and eliminating such artifacts [5,6]. Methanol fixation offers additional benefits, including improved sample integrity and enhanced reproducibility across various bacterial species. Despite these findings, many existing automated systems continue to rely on heat fixation, necessitating further innovation to integrate superior fixation methods into compact, user-friendly devices that can be utilized at the point of care. This underscores the need for technological advancements to refine the Gram staining process while maintaining its accessibility and affordability.

Recent advancements in artificial intelligence (AI) and machine learning have transformed microbiological diagnostics by enabling the automated interpretation of Gram-stained slides. AI-based systems can analyze microscopic images with high precision, improving diagnostic accuracy, standardization, and reproducibility, while reducing the dependency on expert personnel [7,8,9,10]. Such advancements are particularly beneficial in regions with a shortage of trained microbiologists, enabling more consistent and objective results. Additionally, AI-powered solutions can provide rapid preliminary analyses, flagging potential pathogens for further investigation and supporting timely clinical decision-making. However, it is important to note that the full benefits of these advances are contingent upon the availability of a portable Gram stainer, which is a crucial component in the implementation of these automated diagnostic systems.

The present study introduces the Point-of-Care Gram Stainer (PoCGS^®^, CarbGeM Inc. 1-5-13 Jinnan, Shibuya-ku, Tokyo, Japan), a compact, automated system designed specifically for single-slide processing at the point of care. Unlike existing high-throughput machines, PoCGS^®^ incorporates expert-recommended practices such as methanol fixation and precise control over staining parameters to ensure high-quality results. This study aims to evaluate the performance of PoCGS^®^ in comparison to manual staining conducted by skilled experts, focusing on its accuracy, reproducibility, and applicability in diverse clinical environments. By addressing the challenges of portability, ease of use, and slide quality, PoCGS^®^ aims to democratize access to rapid Gram staining, enabling timely and effective infectious disease management in a variety of healthcare settings.

In summary, the evolution of Gram staining from manual methods to automated systems represents a significant step forward in microbiological diagnostics. However, there remains a pressing need for compact, efficient, and cost-effective solutions that can bridge the gap between high-volume laboratories and resource-limited settings. The introduction of PoCGS^®^ marks a crucial development in this landscape, offering a promising solution that combines automation with advanced fixation techniques and AI-powered analysis to revolutionize point-of-care diagnostics.

## 2. Materials and Methods

**Design of the Point-of-Care Gram Stainer (PoCGS^®^)**

We aimed to replicate the technique used by experts when performing Gram staining. For example, experts typically hold the slide glass with one hand and slowly apply staining reagents with the other, ensuring meticulous coverage of the entire sample area.

**Figure 1:**
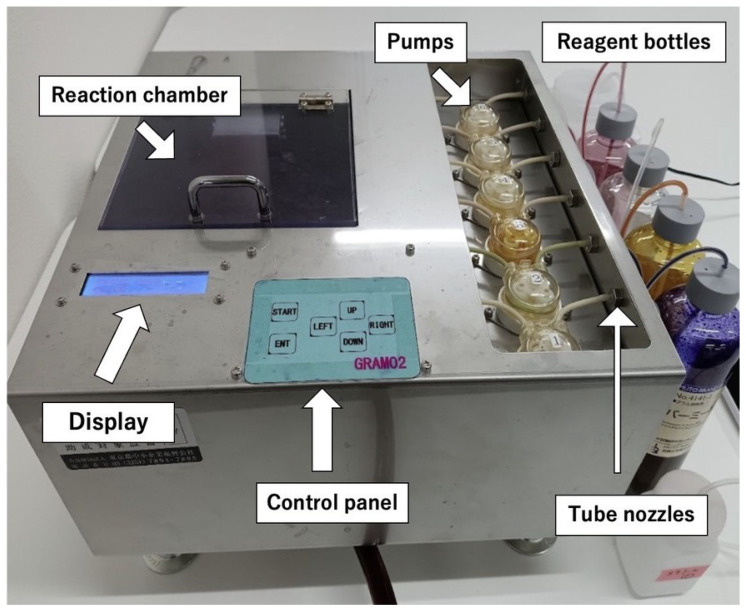
Prototype of PoCGS^®^, Point of Care Gram Stainer.

Figure 1 shows the working prototype of PoCGS^®^, which was used for the subsequent experiments. The prototype measures 320 mm in width, 320 mm in depth, and 210 mm in height (including adjustment devices). For optimal staining results, the prototype must be installed completely horizontally using the adjustment devices and a spirit level. The total weight of the prototype is 4.5 kg (excluding reagents and water).

**Figure 2:**
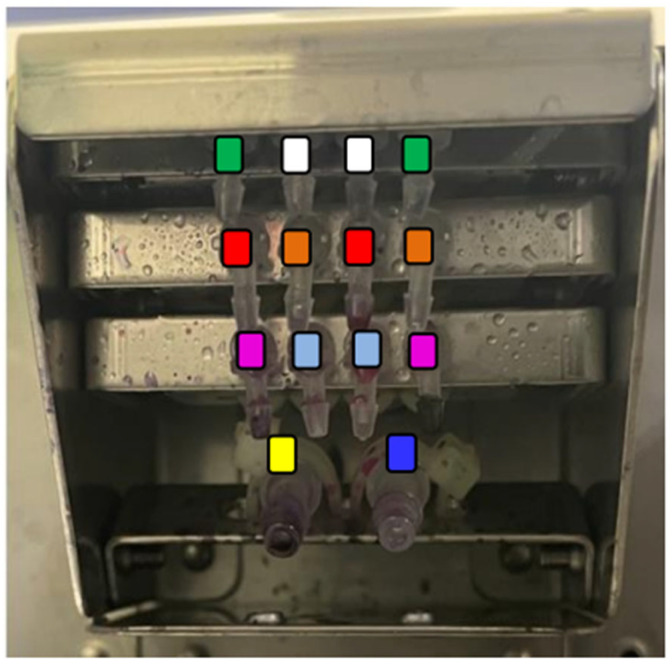
Arrangement of 14 nozzle tips. White: methanol, Purple: crystal violet, Brown: iodine, Light blue: acetone/ethanol, Red: Fuchsin, Green: unused, Blue: rising water, Yellow: blower.

PoCGS^®^ has a slide holder that sits horizontally in the reaction chamber. Rather than employing a moving mechanism to simulate expert hand movements, which would complicate the design, PoCGS^®^ uses six pairs of fixed reagent application nozzles arranged in a 4 × 3 array and placed vertically above one end of the holder (Figure 2). The methanol application nozzles are positioned in the center of the first row (white) to prevent sample removal. Crystal violet nozzles are located at either end of the third row (purple) to ensure full coverage of the sample area. The positions of the other nozzles are less critical: two pairs in the second row (brown and red) are used for iodine and fuchsin; the two inner nozzles in the third row (light blue) are used for the decolorizer (acetone/ethanol); and the two outer nozzles in the first row (green) are reserved for future use. The rinse water and blower use larger nozzles in the last row and are located underneath and nearly horizontal to the holder for effective blowing and rinsing. Each pair of nozzle tips is connected to a specific programable pump, which is connected to the specific reagent summarized in Table 1. Pharmed BPT tubing (Saint-Gobain K.K. Inc. Tour Saint-Gobain—La Défense 12 Place de l‘Iris 92400 Courbevoie 92400 Courbevoie France )is used for reagents, while silicone tubing is used for the rinse water and blower.

The slide glass holder and all the 14 nozzles are equipped in the reaction chamber. The reaction chamber has a sloped bottom surface that allows waste fluid to collect in a central hole. From the central hole, waste fluid can flow through a waste fluid tube into a dedicated waste bottle or sink. With a programmable pump connected to each reagent, the application time (pump run time) and reaction time (wait time) for each reagent, as well as the rinsing time and blow time, can be programmed using the control panel and the display. A sequence of these times constitutes a recipe for a particular sample, and a total of 10 recipes are stored. Users can select one of their favorite or appropriate recipes for specific samples.

After a specimen is smeared on a slide glass, the slide glass is placed onto the holder in the reaction chamber. Once the START button on the Control Panel is pressed, the remaining steps, including methanol fixation, are carried out automatically.

Table 2 summarizes typical parameters such as the reagent application time, reaction time, and blow time. All of these can be customized. The total staining process typically takes 270 s (4 min and 30 s).

**Figure 3:**
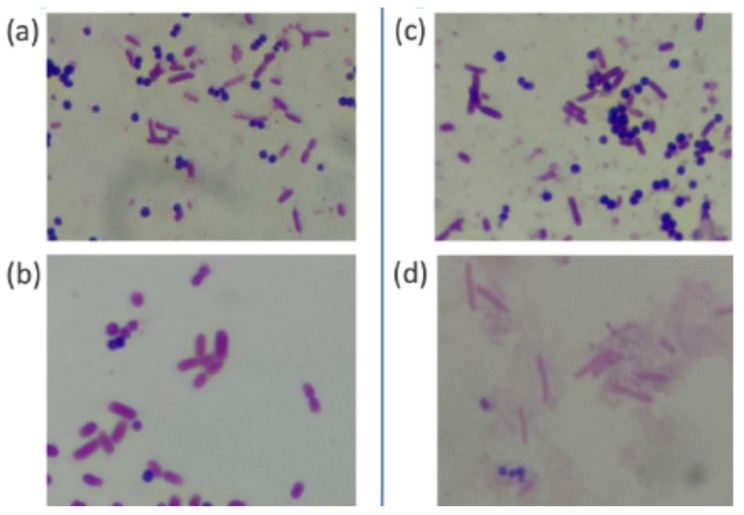
The images of the samples stained by PoCGS^®^ (**a**,**b**) and by the experts (**c**,**d**). Each image clearly shows Gram-negative rods (*E. coli*) and Gram-positive coccus (*Staphylococcus aureus*).

**Figure 4:**
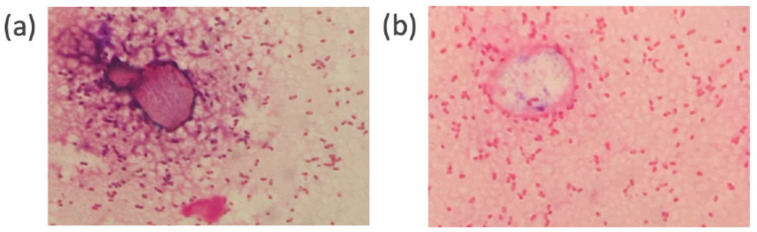
(**a**) A sample image with poor decolorization by PoCGS^®^ showing crystal violet pigment remaining in some of the bacteria and the background. (**b**) A sample image stained by an expert showing the uniform staining of bacteria and background.

Before conducting the comparative study, we performed a preliminary staining test using *E. coli* and *S. aureus* on the same slide. Figure 3 shows images of the samples stained by PoCGS^®^ (a, b) and by experts (c, d). Each image clearly demonstrates Gram-negative rods (*E. coli*) and Gram-positive cocci (*Staphylococcus aureus*). Figure 4a shows an example of poor decolorization by PoCGS^®^, with crystal violet pigment remaining on some bacteria and in the background. Figure 4b shows a sample stained by an expert, demonstrating uniform staining.

**Ethics**

The following tests were conducted at the Department of Clinical Laboratory, Kobe University Hospital (Kobe, Japan), with approval from the Institutional Review Board of Kobe University (IRB registration number: B220163).

**Microbiological Samples**

Clinical urine samples with Gram stain requests were included in the study. A 10 μL aliquot of urine was evenly spread on slides, covering an area of 7.6 cm × 2.6 cm without concentration.

As type strains, *Escherichia coli* (NBRC 15034) and *Staphylococcus aureus* (NBRC 14462) were obtained from the Biological Resource Center, National Institute of Technology and Evaluation (Tokyo, Japan), and used for artificial samples as well.

**Gram Staining**

For Gram staining reagents, we used Neo-B&M Wako products available from FUJIFILM Wako Pure Chemical Corporation (Osaka, Japan). Manual staining followed the protocol of the Department of Clinical Laboratory, Kobe University Hospital. The key time differences between PoCGS^®^ and manual staining are listed in Table 3 with notably shorter reaction times for crystal violet, iodine, and carbol fuchsin in PoCGS^®^, resulting in an overall shorter processing time.

**Manual Staining**

The comparative study included 20 clinical samples and 20 artificial samples (10 *E. coli*, 10 *S. aureus*). Clinical urine specimens were selected for turbidity.

Each specimen was stained by PoCGS^®^, four skilled Gram staining experts, and four untrained medical personnel. Each person stained five clinical and five artificial samples. The staining results were independently evaluated by an expert in Gram staining who did not participate in the staining process.

The primary outcome was the concordance between PoCGS^®^ and expert interpretations, which included classifications such as Gram-positive cocci (GPC), Gram-positive rods (GPR), Gram-negative cocci (GNC), Gram-negative rods (GNR), yeasts, and “no organisms seen”. Slides stained by experts served as the reference standard.

Secondary outcomes were used to evaluate the slide quality based on the following: (1) the uniform staining of organisms, (2) uniform background staining, (3) the absence of staining artifacts (e.g., crystal violet precipitates), and (4) agreement between the microscopy and culture results. Each criterion was scored 0 (absent) or 1 (present), with a maximum total score of 4. Discrepancies between the culture and microscopy were categorized as either major errors (smear negative/culture positive) or minor errors (smear positive/culture negative).

Clinical laboratory technicians who did not participate in Gram staining conducted the evaluation.

**Microscopy**

Expert evaluators examined the slides using a light microscope (OLYMPUS CX-41 with PlanC N 100 ×/1.25 oil FN22, 3-1 Oaza-Odakura-Aza-Okamiyama, Nishigo-mura, Nishishirakawa-gun, Fukushima 961-8061, Japan) at 1000× magnification under oil immersion.

## 3. Results

Of the 40 samples examined, 16 GPC, 20 GNR, 3 yeast, and 2 samples were identified as no organism by PoCGS and the experts. One sample contained both GPC and GNR. Table 4 summarizes the primary outcomes between PoCGS^®^ and skilled experts from 40 staining procedures. For the primary outcome, the interpretations of the slides stained by PoCGS were compared with those by skilled experts. All the slides prepared by PoCGS yielded interpretation results that were completely identical to those prepared by the experts. Since one clinical sample contained both Gram-positive cocci and Gram-negative rod, the total number of cases was 41. GPC was not isolated from this urine sample by aerobic culture.

Table 5 summarizes the secondary outcomes between PoCGS^®^, skilled experts (A1–A4), and unskilled medical personnel (B1–B4). The secondary outcomes were (1) the uniform staining of bacteria/fungi, (2) the uniform staining of the background, (3) no staining artifacts, and (4) agreement between the culture results and microscopy examination. Each criterion was scored as 0 (absent) or 1 (present) to calculate a quality score with a maximum possible score of 4 for each sample. With 4 criteria and 40 samples, the total score for each stain was 160. The average score was the total score divided by the number of samples, which was 40.

Skilled experts (A1–A4) generally provided more consistent and higher ratings compared to unskilled personnel (B1–B4). The average scores of the skilled and unskilled personnel were 3.825 (*p* < 0.001) and 3.625 (*p* < 0.001), respectively. PoCGS^®^ shows a relatively consistent performance across various strains, often matching the quality ratings given by the experts; however, the average score of PoCGS was 2.875, lower than both the skilled and unskilled personnel.

In detail, PoCGS^®^ was comparable regarding (3) no staining artifacts and (4) agreement between the culture results and microscopy examination.

Especially regarding (4), PoCGS^®^ showed comparable staining performance not only on *E. coli* and *S. aureus*, but also on three other GNRs, four other GPCs, and four yeasts. However, PoCGS^®^ was inferior to the manual staining regarding (1) the uniform staining of bacteria/fungi and (2) the uniform staining of the background.

## 4. Discussion

We evaluated the quality of the Point-of-Care Gram Stainer (PoCGS^®^) and compared the results with the manual staining performed by skilled experts and untrained medical personnel. Most importantly, there was no difference in the microscopic interpretation of slides between those stained by PoCGS^®^ and those prepared by skilled experts. Although Gram-positive rods (GPR) and Gram-negative cocci (GNC) were not included in this particular analysis, the accurate staining of Gram-positive cocci (GPC) and Gram-negative rods (GNR) suggests that PoCGS^®^ is capable of handling these additional morphologies without issue. Given the perfect concordance with expert staining interpretations, PoCGS^®^ provides a reliable alternative for Gram stain interpretation and has potential utility in clinical practice for protocol standardization and rapid diagnosis.

The quality of slides stained by PoCGS^®^ was not as good as those stained by manual stain by skilled and unskilled medical personnel. Specifically, while PoCGS^®^ produced comparable results in terms of artifact minimization and agreement with the culture outcomes—including four GNRs, five GPCs, and four yeast identifications (Table 5)—it underperformed in two key areas: (1) the homogeneous staining of microorganisms and (2) effective negative staining of the background. These limitations, however, did not affect the personnels’ ability to identify bacterial species. This difference in staining quality may be attributed to PoCGS^®^ applying a uniform protocol with fixed timing, whereas manual staining allows experts to adjust the decolorization duration based on the specimen thickness. To address this issue, PoCGS^®^ protocols can be customized by modifying the duration of reagent application and the drying (blowing) phase. These parameters are likely crucial to achieving more homogeneous and effective staining and decolorization.

For this study, we used turbid urine samples as clinical specimens. According to the guidelines of the Japanese Association of Medical Technologists (JAMT), urine appearance is evaluated based on five criteria: color, turbidity, macrohematuria, and the presence of foreign bodies. Turbid urine is considered likely to contain higher bacterial loads and is often associated with pyuria.

Macrohematuria, on the other hand, may be caused by cystitis, urinary tract malignancy, or renal disease. At Kobe University Hospital, hematuric urine samples are first used for urinalysis and sediment examination before microbiological testing and were therefore excluded from our study. This decision was based on the concern that a high concentration of red blood cells in macrohematuria could interfere with the quality of Gram staining.

Our findings suggest that PoCGS^®^ is a promising tool for the point-of-care diagnosis of urinary tract infections via Gram staining. However, Gram staining is also routinely applied to other types of clinical specimens. For example, Gram stain of sputum samples can guide antibiotic therapy and assist in the selection of appropriate antimicrobials for managing ventilator-associated pneumonia. Future studies should explore the applicability of PoCGS^®^ to other clinical sample types to further validate its utility across broader diagnostic settings [11,12,13,14,15,16,17]. Furthermore, sputum Gram stain is not only a point-of-care test used for diagnosis, but might be able to monitor the occurrence of ventilator-associated pneumoniae [2,18,19,20].

The role of body fluid Gram staining extends beyond diagnostic purposes; it also serves as a valuable parameter for monitoring the effectiveness of treatment. In managing bacterial infections, clinicians typically assess antibiotic efficacy using various clinical indicators, such as symptom resolution, the recovery of blood pressure, respiratory rate, body temperature, imaging findings, and laboratory results. However, these indicators often lack organ specificity and can vary depending on the patient’s overall condition. For instance, if the patient is not alert or unable to communicate, symptom-based assessments become unreliable. Additionally, if the patient has multiple concurrent infections, systemic inflammatory markers—such as their white blood cell count, C-reactive protein (CRP), or erythrocyte sedimentation rate (ESR)—may be elevated regardless of their response to treatment in a specific organ.

From this perspective, the Gram staining of body fluid samples collected directly from the infected site may offer a more precise, organ-specific method for evaluating treatment efficacy. Looking ahead, we plan to expand the application of automated Gram staining to other types of clinical specimens, including sputum and pus. Maintaining high-quality staining in these samples will require precise control of the decolorization step, which must be adjusted according to the specimen’s thickness and composition. As PoCGS^®^ is currently a prototype, its long-term durability has not yet been established. We plan to evaluate the performance and reliability of the production model in the near future.

Finally, laboratory automation and workflow optimization—enabled by tools like PoCGS^®^—can play a significant role in reducing the turnaround time of microbiological results, ultimately supporting faster and more effective clinical decision-making [21]. PoCGS^®^ can reduce the Gram staining time and contribute in this situation. We also plan to compare the turnaround time of the Gram stain results between PoCGS^®^ staining and manual staining in the night float situation. PoCGS^®^ can contribute to reducing the turnaround time of reportable positive blood culture results and pathogens.

Finally, we plan to integrate the AI-based interpretation of Gram-stained slides into the PoCGS^®^ system to further enhance its potential by providing immediate feedback to support faster clinical decision-making and improve patient outcomes.

## Figures and Tables

**Table 1:** Assignment of each nozzle tip to pumps.

Nozzle Color	Pump	Reagent
white	P1	methanol
purple	P2	crystal violet
brown	P3	iodine
light blue	P4	acetone/ethanol
red	P5	fuchsin
green	unused	optional
blue	P6	rinsing water
yellow	Blower	(air)

**Table 2:** The typical staining parameters for PoCGS^®^.

	Application Time (s)	Reaction Time (s)
Methanol fixation	5	60
Blower	5	0
Crystal violet	10	30
Water rinse	10	0
Blower	3	0
Iodine	10	0
Water rinse	10	0
Blower	3	0
Decolorizer	6	45
Water rinse	10	0
Blower	3	0
Carbol Fuchsin	10	30
Water rinse	10	0
Blower	10	0
Total	105	165

**Table 3:** Difference in staining parameters between manual and PoCGS^®^.

Step	Time in Manual Staining (s)	Time in PoCGS^®^ (s)
Crystal violet	60	30
Iodine	60	0
Decoloration	Until color removal is complete	45
Carbol fuchsin	60	30

**Table 4:** Summary of the comparison study between PoCGS^®^ and skilled experts in the primary outcome, and the concordance of the interpretation with 40 samples examined.

Stainer	Interpretation						
	GPC	GPR	GNR	GNC	Yeast	No Organism	Total
PoCGS	16	0	20	0	3	2	41
Experts	16	0	20	0	3	2	41
Concordance	100%	N/A	100%	N/A	100%	100%	100%

Note: Since one clinical sample contained both Gram-positive cocci and Gram-negative rods, the total number of cases was 41. N/A: GPR and GNC were not detected in the sample.

**Table 5:** Comparison of quality of Gram stain between PoCGS^®^, skilled experts (A1–A4), and unskilled medical personnel (B1–B4). Number 1 to 20 are clinical samples and 21–40 are artificial samples.

Sample Number	Culture Result or Standard Strains	Category	A1	A2	A3	A4	B1	B2	B3	B4	PoCGS
1	*S. agalactiae*	GPC	4				4				3
2	*E. coli*	GNR	4				4				4
3	*P. aeruginosa*	GNR	4				4				4
4	*S. agalactiae*	GPC	4				3				3
5	*E. faecalis*	GPC	4				3				3
6	*S. aureus*	GPC		4				4			3
7	*C. albicans*	Yeast		3				4			3
8	*E. coli*	GNR		4				3			4
9	*S. haemolyticus* *S. epidermidis*	GPC		3				4			3
10	*E. coli*	GNR GPC		4				4			4
11	No growth	No growth			3				4		1
12	*E. coli*	GNR			4				3		3
13	*C. parapsilosis*	Yeast			4				3		3
14	*K. pneumoniae*	GNR			4				4		2
15	*P. aeruginosa*	GNR			3				3		3
16	No growth	No growth				3				4	2
17	*P. aeruginosa*	GNR				4				4	2
18	*S. marcescens* *P. aeruginosa*	GNR				4				4	3
19	*C. tropicalis* *C. albicans*	Yeast				4				3	3
20	*P. mirabilis*	GNR				3				3	3
21	*S. aureus* (NBRC 14462)	GPC	4				3				3
22	*S. aureus* (NBRC 14462)	GPC	4				4				4
23	*E. coli* (NBRC 15034)	GNR	4				4				4
24	*S. aureus* (NBRC 14462)	GPC	4				3				3
25	*E. coli* (NBRC 15034)	GNR	4				4				3
26	*E. col i*(NBRC 15034)	GNR		4				4			3
27	*S. aureus* (NBRC 14462)	GPC		4				4			3
28	*S. aureus*(NBRC 14462)	GPC		4				4			4
29	*E. coli* (NBRC 15034)	GNR		4				3			3
30	*S. aureus* (NBRC 14462)	GPC		4				4			4
31	*S. aureus* (NBRC 14462)	GPC			4				3		1
32	*E. coli* (NBRC 15034)	GNR			4				4		3
33	*S. aureus* (NBRC 14462)	GPC			4				3		3
34	*E. coli* (NBRC 15034)	GNR			3				4		2
35	*E. coli* (NBRC 15034)	GNR			4				3		3
36	*E. coli* (NBRC 15034)	GNR				4				4	2
37	*S. aureus* (NBRC 14462)	GPC				4				4	2
38	*S. aureus* (NBRC 14462)	GPC				4				3	3
39	*E. coli* (NBRC 15034)	GNR				3				4	3
40	*E. coli* (NBRC 15034)	GNR				4				4	3
The average score for each expert/non-expert/PoCGS			4	3.8	3.7	3.7	3.6	3.8	3.4	3.7	3.0
The average score for grouped experts and non-experts			3.8				3.6				

Note: Gram stain of sample 10 revealed both GPC and GNR, whereas the aerobic culture revealed only *E. coli*.

## Data Availability

All the data are included in this manuscript.
